# Potential of three probiotic lactobacilli in transforming star fruit juice into functional beverages

**DOI:** 10.1002/fsn3.775

**Published:** 2018-09-12

**Authors:** Yuyun Lu, Chin‐Wan Tan, Dai Chen, Shao‐Quan Liu

**Affiliations:** ^1^ Food Science and Technology Program Department of Chemistry National University of Singapore Singapore city Singapore; ^2^ Beijing Key Laboratory of Viticulture and Enology College of Food Science and Nutritional Engineering China Agricultural University Beijing China; ^3^ National University of Singapore (Suzhou) Research Institute Jiangsu China

**Keywords:** *Averrhoa carambola*, *Lactobacillus*, probiotics, star fruit

## Abstract

The star fruit is popularly cultivated and consumed in Southeast Asia due to its high antioxidant capacity and various nutrients. In this study, three commercial probiotic strains (*Lactobacillus helveticus* L10, *Lactobacillus paracasei* L26, and *Lactobacillus rhamnosus* HN001) were evaluated in star fruit juice fermentation and all strains grew well with the final cell counts of around 10^8^ CFU/ml. The star fruit juice fermented by *L. rhamnosus* produced the highest amount of lactic acid, resulting in a significant lower pH (4.41) than that of *L. helveticus* (4.76) and *L. paracasei* (4.71). Most of aldehydes and esters endogenous in star fruit juice decreased to low or undetectable levels, while ketones, alcohols, and fatty acids were produced at varying levels that could impart different aroma notes to the beverages. Therefore, the selection of appropriate probiotics can be an alternative way to develop new functional beverages from star fruit juice with specific aroma notes.

## 1 INTRODUCTION

Carambola (or star fruit) is the fruit of the *Averrhoa carambola* tree and is one of the most popular and widely cultivated fruits in Southeast Asia. It consists of five prominent longitudinal ridges, which give rise to its unique and attractive star‐shaped cross section. Stat fruit comprises of various nutrients (carbohydrates, proteins, amino acids, and minerals) and is rich in proanthocyanidins, epicatechin, and vitamin C, which provide a myriad of health benefits to humans (Shui & Leong, 2006).

Star fruit is normally consumed fresh or is used to produce jellies, sweets, and cordial concentrates due to its highly perishability especially in tropical regions (e.g., Singapore, Malaysia, and Indonesia). Thus, preservation methods and processing procedures such as modified atmosphere packaging and drying process have been developed for star fruits (Teixeira, Durigan, Alves, & O'Hare, 2007).

Fermentation is a biotechnological process that can be employed to promote the valorization of sustainability of star fruits as well as enhance its nutritional or functional properties. Star fruit juice has served as an alternative material to produce fruit vinegar and wine (Chandra, 2010; Chang, Lee, & Ou, 2005). Therefore, it is possible that star fruit juice may also be fermented into probiotic beverages with enhanced functional benefits.

Probiotics are live microorganisms, which when administered in adequate amounts confer a health benefit to the host according to FAO (2001). Bifidobacteria and lactobacilli are the most commonly used probiotics in fermented dairy products. To date, probiotic strains that have been isolated and widely used in commercial products include *Lactobacillus acidophilus*,* Lactobacillus casei*,* Lactobacillus rhamnosus,* and *Bifidobacterium bifidum* (Heller, 2001). Studies have shown that *L. casei* could help to prevent enteric infections and stimulate immune responses in an animal model (Perdigon, Alvarez, Rachid, Agüero, & Gobbato, 1995), while supplementation of *L*. *rhamnosus* HN001 enhanced immunity in the elderly people (Gill, Rutherfurd, & Cross, 2001). In addition, *L. acidophilus* and *L. casei* could promote cellular cholesterol reduction (Lye, 2010), and the important roles of *L. rhamnosus* GG and *L. casei* in prevention and treatment of pediatric diarrhea have also been well studied (Nixon, Cunningham, Cohen, & Crain, 2012; Wanke & Szajewska, 2014). Furthermore, the beneficial effects of lactobacilli on oral health (e.g., the reduction in dental caries incidences and salivary mutan formation) are also documented (Campus et al., 2014).

Probiotics are mostly found in yoghurt and fermented milks, because they are known to be excellent carriers for probiotics due to their good buffering capacity. However, consumers who suffer from lactose intolerance may not be able to enjoy the benefits of probiotic dairy products (Hertzler, Dennis, Jackson Karry, Bhriain, & Suarez, 2013). Therefore, nondairy probiotic beverages such as probiotic fruit juices would serve as an alternative for such consumers.

Of late, Lee, Boo, and Liu (2013) and Lu, Putra, and Liu (2018) have reported the successful probiotic fermentation (using *L. acidophilus* and *L. casei*) in coconut water and durian pulp, respectively. The probiotic fermentation contributed unique flavor profiles to these fruit juices, which further raises interest in studying such fruit juices. However, the relatively low pH of fruit juices (<4) would pose a challenge to the growth of probiotics in such acidic media (Nagpal, Kumar, & Kumar, 2012). To enhance the survival and sustainability of probiotic lactobacilli in acidic juices, adjustment of pH of the media and selection of a robust strain could be conducted (Sheehan, Ross, & Fitzgerald, 2007). Therefore, the aim of this study was to evaluate the growth and metabolism of three probiotic lactobacilli (*Lactobacillus helveticus* L10, *Lactobacillus paracasei* L26, and *L. rhamnosus* HN001) at 30°C (prevalent in tropical countries). Ultimately, the intent is to develop a novel probiotic fermented functional star fruit juice beverage.

## 2 MATERIALS AND METHODS

### 2.1 Star fruit juice preparation

Star fruits were purchased from a local supermarket in Singapore. Skin and seeds were removed from the pericarp before juicing in a blender. The crude juice was then centrifuged and filtered using a muslin cloth to remove the suspended solids. The initial total soluble solids content (°Brix) and pH were 7.09 and 3.58, respectively. The pH of the star fruit juice was adjusted to 5.9 (1 mol/L NaOH) to enable growth of lactobacilli. The star fruit juice was then filter‐sterilized by sequentially passing through a 0.65‐μm and 0.45‐μm polyethersulfone filter membrane aseptically.

### 2.2 Probiotic strains and preculture preparation

Three probiotic strains including *L. helveticus* (formerly *acidophilus*) L10 and *L. paracasei* L26 (both from Lallemand, Montreal, Canada) and *L. rhamnosus* HN001 (DuPont‐Danisco, Singapore) were used in this study. The freeze‐dried pure cultures were propagated in respective MRS broth at 37°C for 48 hr. The pure cultures were then stored at −80°C before use.

The precultures of probiotic strains were prepared separately by inoculating 10% (v/v) of the respective pure cultures into sterile star fruit juice. This was then followed by incubation at 37°C for 48 hr to achieve the cell forming unit (CFU) at least 10^7^ per ml.

### 2.3 Fermentation of lactobacillus strains in start fruit juice

Triplicate fermentations were conducted by inoculating 1% (v/v) precultures of each probiotic strain into 250 ml of sterile star fruit juice in 500‐ml conical flasks. The fermentation was then incubated at 30°C for 8 days. Samples were taken at Days 0, 1, 2, 4, 6, and 8 for chemical and microbiological analyses under aseptic condition.

### 2.4 Analytical determinations

The pH was measured using a pH meter (Metrohm, Herisau, Switzerland), and °Brix was determined by a refractometer (ATAGO, Yushima, Japan), respectively. The viable cell counts of *Lactobacillus* strains were determined by plating on MRS agar (62 g/L; Oxoid, Basingstoke, UK) and incubated at 37°C for 48 hr before plate counting.

High‐performance liquid chromatography (HPLC) (Shimadzu, Kyoto, Japan) was used for the determination of sugars and organic acids. The separation and detection of sugars were performed using a Zorbax carbohydrate column (Agilent, Santa Clara, CA USA) with a low temperature evaporative light scattering (ELSD‐LT) detector (40°C, 350 kPa N_2_). An isocratic flow rate of 1.4 ml/min was used for mobile phase consisting of acetonitrile–water mixture (80:20 v/v). The organic acids were analyzed using a Supelcogel C‐610H column (300 × 7.8 mm, Supelco, Sigma‐Aldrich, Barcelona, Spain) connected to a SPD‐M20A photodiode array detector at 210 nm (Shimadzu, Kyoto, Japan). The column was eluted with sulfuric acid (0.1% v/v) as the mobile phase at 40°C and 0.4 ml/min flow rate. Calibration curves were established for all analyzed compounds with *R* ^2^ > 0.99. Prior to injection, samples were centrifuged at 20,379 *g* for 15 min at 4°C, followed by filtration using a 0.20‐μm regenerated cellulose filter membrane (Sartorius Stedim Biotech, Gottingen, Germany).

Headspace solid‐phase microextraction (SPME) sampling was combined with gas chromatography (GC)‐mass spectrophotometer (MS) and flame ionization detector (FID) for qualitative analysis of the volatiles as described by Lee, Ong, Yu, Curran, and Liu (2010). The star fruit juice was adjusted to pH 2.5 with 1 mol/L HCl, and 5 ml of the sample was transferred to a 20‐ml glass headspace vial sealed with a polytetrafluoroethylene septum. The extraction of volatiles was performed by a SPME autosampler (CTC, Combi Pal, Switzerland) using a carboxen/polydimethylsiloxane fiber (85 μm film thickness, Supelco, Sigma‐Aldrich, Barcelona, Spain). Sample was subjected to 250 rpm agitation at 60°C for 45 min. This was followed by thermal desorption of the SPME fiber at 250°C in the injection port of an Agilent 7890A gas chromatograph coupled to an Agilent 5975C triple‐axis MS and FID (Santa Clara, CA, USA). Separation of volatiles was carried out in an oven temperature programmed from 50°C (5 min) to 230°C (30 min) at 5°C/min, by a capillary column coated with 0.25 μm polyethylene glycol film modified with nitroterephthalic acid (60 m × 0.25 i.d., Agilent DB‐FFAP, Santa Clara, CA, USA), where helium gas was used as the carrier gas at a linear flow rate of 1.2 ml/min. The Wiley 275 and mass spectral databases were used for identification by matching the mass spectral of the volatiles. The linear retention indices (LRI) of the compounds were used to further confirm the results. Retention times of the samples and standard compounds (alkanes, C8‐C40) run under same conditions were used for the calculation of LRI values, as shown in following equation:

$$\text{LRI}=100\times \left(\frac{t-t_{n}}{t_{n+1}-t_{n}}+n\right)$$

where *t* represents the retention time of interest compounds in min, *n* is the number of carbon atoms of the n‐alkane eluting before the compound; whereas *t*_*n*_ and *t*_*n*+1_ are the retention time of the alkanes eluting before and after the interest compound, respectively.

### 2.5 Statistical analysis

All analyses were carried out based on the data from the triplicate fermentations. One‐way analysis of variance (ANOVA) and Scheffe's test were performed using SPSS 19.0 (Statistical Program for Social Sciences, SPSS Corporation, Chicago, IL), and significant difference was evaluated at the 95% confidence interval. Principal component analysis (PCA) was performed using software MATLAB R2008a (MathWorks, Natick, MA, USA) to analyze the distribution of aroma profiles of star fruit juice and star fruit juice beverages fermented with different probiotic strains.

## 3 RESULTS AND DISCUSSION

### 3.1 Growth of three probiotic strains

**Figure 1:**
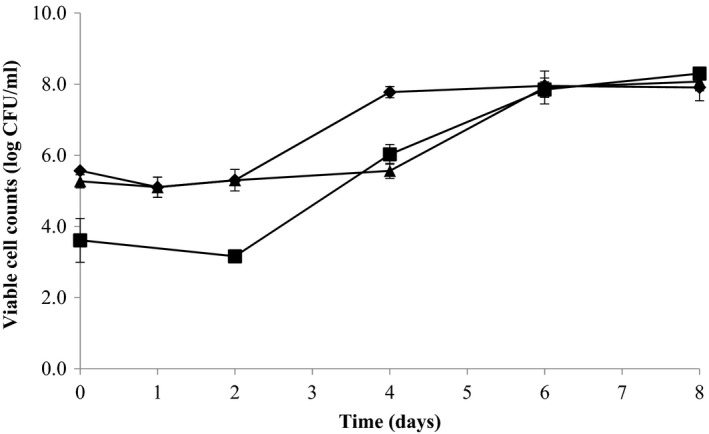
Kinetic changes in three probiotic strains during star fruit juice fermentation. *Lactobacillus helveticus* L10 (■); *Lactobacillus paracasei* L26 (▲); *Lactobacillus rhamnosus* HN001 (♦)

The growth of three lactobacilli strains in star fruit juice is shown in Figure 1. *Lactobacillus paracasei* exhibited a longer lag phase (4 days), whereas *L. helveticus* and *L. rhamnosus* increased rapidly after 2 days of the lag phase (Figure 1). *Lactobacillus rhamnosus* increased to 6.23 × 10^7^ CFU/ml on Day 4, while the other two strains were able to reach similar cell counts on Day 6 (Figure 1). After which, *L. rhamnosus* and *L. paracasei* entered the stationary phase on Day 4 and Day 6, respectively. On the other hand, although *L. helveticus* started from a lower cell count (8.50 × 10^3^ CFU/ml) compared with the other two strains (~10^5^ CFU/ml), it was able to increase to 7.30 × 10^7^ CFU/ml on Day 6 and continued to grow to 2.07 × 10^8^ CFU/ml on Day 8 (Table 1).

**Table 1:** Parameters of star fruit juice (Day 0) and star fruit juice beverages (Day 8) fermented by *Lactobacillus helveticus* L10, *Lactobacillus paracasei* L26, and *Lactobacillus rhamnosus* HN001

	Star fruit juice (Day 0)	Star fruit beverages (Day 8)		
		L10	L26	HN001
^o^Brix	7.09 ± 0.01^a^	6.97 ± 0.03^b^	6.98 ± 0.03^b^	6.93 ± 0.01^b^
pH	5.91 ± 0.01^a^	4.76 ± 0.11^b^	4.71 ± 0.03^b^	4.41 ± 0.02^c^
Viable cell count (10^8^ CFU/ml)	*	2.07 ± 0.76^a^	1.30 ± 0.65^a^	1.05 ± 0.96^a^
Sugars (g/L)				
Fructose	29.0 ± 1.0^a^	29.1 ± 6.8^a^	30.4 ± 0.5^a^	30.2 ± 1.3^a^
Glucose	30.8 ± 0.9^a^	30.1 ± 1.2^a^	31.6 ± 0.2^a^	31.7 ± 0.6^a^
Sucrose	7.0 ± 0.2^a^	0.0 ± 0.0^b^	0.0 ± 0.0^b^	0.0 ± 0.0^b^
Organic acids (g/L)				
Acetic acid	0.03 ± 0.01^a^	0.28 ± 0.01^b^	0.04 ± 0.00^a^	0.03 ± 0.00^a^
α‐Ketoglutaric acid	0.00 ± 0.00^a^	0.06 ± 0.01^a^	0.06 ± 0.00^a^	0.06 ± 0.00^a^
Citric acid	0.15 ± 0.00^a^	0.14 ± 0.00^ab^	0.14 ± 0.00^b^	0.14 ± 0.00^b^
Lactic acid	0.00 ± 0.00^a^	3.43 ± 0.11^b^	3.70 ± 0.09^b^	4.40 ± 0.23^c^
Malic acid	3.54 ± 0.02^a^	2.04 ± 0.11^b^	1.98 ± 0.03^b^	1.86 ± 0.10^b^
Oxalic acid	1.47 ± 0.02^a^	1.46 ± 0.01^a^	1.45 ± 0.01^a^	1.43 ± 0.01^a^
Succinic acid	0.72 ± 0.06^a^	0.72 ± 0.16^ab^	0.86 ± 0.03^b^	0.84 ± 0.03^b^

*Notes*

L10: *Lactobacillus helveticus* L10; L26: *Lactobacillus paracasei* L26; HN001: *Lactobacillus rhamnosus* HN001.

^a,b,c^Statistical analysis at 95% confidence level with same letters indicating no significant difference.

*Initial cell counts for strains L10, L26, and HN001 were 7.33 × 10^3^, 1.97 × 10^5^, and 3.69 × 10^5 ^CFU/ml, respectively.

The growth patterns of the three probiotic stains used in this study were not consistent with that observed by Lee et al. (2013), who showed that *L. paracasei* and *L. helveticus* increased to ~10^8 ^CFU/ml within 2 days in coconut water without experiencing a lag phase. The lag phase in this study could be ascribed to the suboptimal fermentation temperature (30°C) and differences in nutrients. This agreed with the findings of Mousavi, Mousavi, Razavi, Emam‐Djomeh, and Kiani (2011), where also reported a lag phase of *L. paracasei* and *L. acidophilus* in pomegranate juice fermentation at 30°C. However, our results were in contrast to some other studies, where lactobacilli could grow rapidly in fruit and vegetable juices at 30°C without going through the lag phase (Wang, Ng, Su, Tzeng, & Shyu, 2009). This could infer that other factors such as growth inhibitors and nutrients availability in the media may also affect the growth of probiotic strains (Siragusa et al., 2014).

Although *L. paracasei* and *L. rhamnosus* were inoculated at similar cell counts (~10^5^ CFU/ml), *L. paracasei* needed a longer time to reach the maximum cell count (6 days) compared to *L. rhamnosus* (Figure 1), indicating that *L. paracasei* was a less robust strain for star fruit juice beverage fermentation. On the other hand, *L. helveticus* showed prolific growth, with a 4‐log increase in the cell population, despite starting off with an initial cell count of only ~10^3^ CFU/ml (Figure 1). Besides, *L. helveticus* reached a final cell count of 1.6‐fold to 2.0‐fold higher than that of *L. paracasei* and *L. rhamnosus*, respectively (Table 1). This indicated that *L. helveticus* could be a better candidate for star fruit juice fermentation.

### 3.2 Changes of °Brix and pH

**Figure 2:**
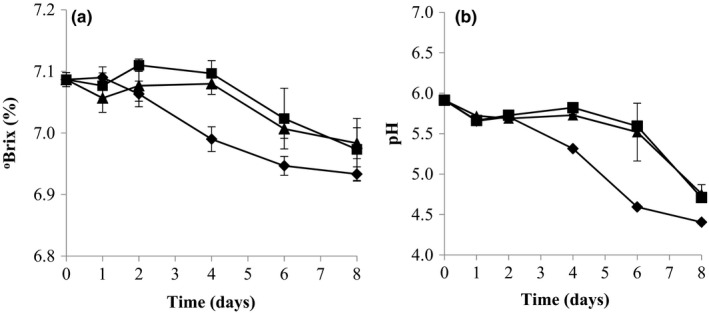
(a) Changes in total soluble solids (°Brix) and (b) pH during star fruit juice fermentation. *Lactobacillus helveticus* L10 (■); *Lactobacillus paracasei* L26 (▲); *Lactobacillus rhamnosus* HN001 (♦)

The changes in °Brix and pH served as indicators to monitor the fermentation progress in star fruit juice. All three probiotic strains resulted in slight decreases in °Brix from 7.09 to around 6.93–6.98 (Figure 2a). On the other hand, the pH values gave a good overview of the fermentation progress. *L. helveticus* and *L. paracasei* shared similar trends of pH changes, in which their pH values decreased slightly from 5.91 on Day 0 to around 5.52–5.60 on Day 6 and then shapely reduced to around 4.71–4.76 on Day 8 (Figure 2b). However, the star fruit juice fermented by *L. rhamnosus* exhibited a different trend of pH changes, where the pH value decreased substantially from 5.71 (Day 2) to 4.60 (Day 6) and then slightly decreased to 4.41 (Day 8), which was significantly lower than the other two strains (Figure 2b). The changes in pH corresponded to the differences in growth, production of lactic acid, and consumption of sugars during fermentation, especially by *L. rhamnosus* relative to the other two lactobacilli (Figure 1, Table 1).

### 3.3 Changes in sugars

**Figure 3:**
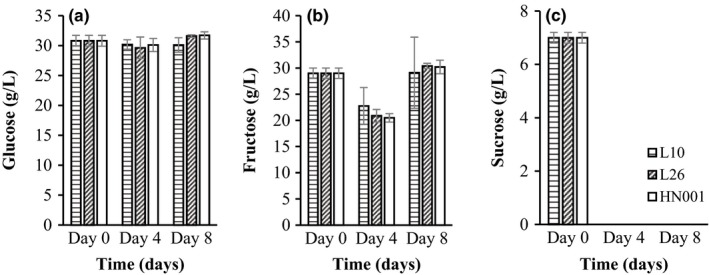
Changes in glucose (a), fructose (b), and sucrose (c) during star fruit juice fermentation

Figure 3 shows sugar utilization by all three probiotic strains. Sucrose was totally depleted, and fructose decreased to 20.5–22.8 g/L on day 4 (Figure 3). Nevertheless, glucose remained unchanged. Our results were in line with findings of Lee et al. (2013). The decrease in sucrose could be ascribed to the acid and/or enzymatic hydrolysis. However, no increase in glucose and fructose was observed during this period despite the decomposition of sucrose, indicating that *Lactobacillus* strains utilized glucose and fructose as their energy sources (Srinivas, Mital, & Garg, 1990) in counterbalance to the formation of glucose and fructose from sucrose hydrolysis.

It is interesting to note that fructose increased from 20.5 to 22.8 g/L (day 4) to 29.4–30.4 g/L on Day 8 in all fermentations (Figure 3). This could be due to the hydrolysis of fructooligosaccharides (FOS) by probiotic strains during fermentation (Kaplan & Hutkins, 2003). FOS is a known prebiotic for probiotics, and 1‐kestose (G‐F2, 1F‐β‐D‐fructofuranosyl‐sucrose) and nystose ([G‐F3, 1F(1‐β‐D‐fructofuranosyl)2 sucrose] have been reported in star fruit (Emanuel, Benkeblia, & Lopez, 2013).

### 3.4 Changes in organic acids

The changes in organic acids in star fruit juice fermentation are shown in Table 1. The slight decrease in citric acid in all fermentations could be ascribed to the citrate fermentation pathway via citrate lyase (Hugenholtz, 1993; Mortera, Pudlik, Magni, Alarcón, & Lolkema, 2013), resulting in the formation of acetic acid and flavor compounds (diacetyl and acetoin) as shown in Table 1 and Table 2, respectively. The star fruit juice fermented with *L. helveticus* (0.28 g/L) produced significantly higher level of acetic acid than that of *L. paracasei* (0.04 g/L) and *L. rhamnosus* (0.03 g/L) (Table 1), possibly due to metabolism of some amino acids such as serine and alanine.

**Table 2:** Major volatile compounds (GC‐FID peak area × 10^6^) and their relative peak areas (RPA) identified in star fruit juice (Day 0) and star fruit juice beverage (Day 8) fermented by *Lactobacillus helveticus* L10, *Lactobacillus paracasei* L26, and *Lactobacillus rhamnosus* HN001

Compounds	LRI^d^	Star fruit juice (Day 0)		Star fruit beverages (Day 8)					
				L10		L26		HN001	
		Peak area	RPA (%)^&^	Peak area	RPA (%)	Peak area	RPA (%)	Peak area	RPA (%)
Acids									
Acetic acid	1459	1.04 ± 0.31^a^	0.63	9.18 ± 6.46^a^	9.43	1.89 ± 0.27^a^	1.43	2.39 ± 1.25^a^	1.69
Hexanoic acid	1845	0.65 ± 0.16^a^	0.39	1.47 ± 0.63^a^	1.51	0.90 ± 0.09^a^	0.68	0.92 ± 0.35^a^	0.65
(*E*)‐2‐Hexenoic acid	–	0.34 ± 0.03^a^	0.20	1.28 ± 0.75^a^	1.31	0.53 ± 0.12^a^	0.40	0.47 ± 0.19^a^	0.33
Decanoic acid	2276	0.48 ± 0.06^a^	0.29	0.64 ± 0.07^a^	0.66	0.48 ± 0.05^a^	0.36	0.48 ± 0.07^a^	0.34
	Subtotal	**2.52**	**1.51**	**12.58**	**12.91**	**3.80**	**2.87**	**4.24**	**3.01**
Alcohols									
Isoamyl alcohol	1210	0.00 ± 0.00^a^	0.00	0.57 ± 0.06^b^	0.58	0.76 ± 0.32^b^	0.57	0.44 ± 0.14^ab^	0.31
1‐Hexanol	–	1.77 ± 0.32^a^	1.07	2.42 ± 0.51^a^	2.49	5.47 ± 0.43^b^	4.13	11.88 ± 0.38^c^	8.40
(*E*)‐2‐Hexen‐1‐ol	1406	3.29 ± 0.16^a^	1.98	6.52 ± 2.39^ab^	6.70	9.97 ± 0.90^bc^	7.53	12.71 ± 2.88^c^	8.98
1‐Octen‐3‐ol	1448	0.00 ± 0.00^a^	0.00	3.84 ± 0.12^b^	3.94	2.92 ± 1.66^b^	2.20	4.63 ± 0.58^b^	3.27
1‐Heptanol	1452	0.38 ± 0.07^a^	0.23	0.34 ± 0.06^a^	0.35	0.49 ± 0.16^a^	0.37	0.45 ± 0.13^a^	0.32
2‐Ethylhexanol	1488	1.08 ± 0.02^a^	0.65	2.25 ± 0.36^ab^	2.31	2.19 ± 0.24^ab^	1.65	2.66 ± 0.77^b^	1.88
Linalool	1542	0.41 ± 0.03^a^	0.25	0.60 ± 0.09^a^	0.62	1.74 ± 0.01^b^	1.31	1.69 ± 0.20^b^	1.20
1‐Nonanol	–	0.88 ± 0.09^a^	0.53	0.90 ± 0.07^a^	0.92	1.14 ± 0.19^a^	0.86	0.92 ± 0.28^a^	0.65
Dihydro‐β‐ionol	1866	0.33 ± 0.03^a^	0.20	0.31 ± 0.07^a^	0.32	0.38 ± 0.03^a^	0.29	0.32 ± 0.03^a^	0.23
	Subtotal	**8.14**	**4.91**	**17.76**	**18.23**	**25.06**	**18.91**	**35.70**	**25.24**
Aldehydes									
1‐Hexanal	1083	5.37 ± 0.51^a^	3.23	0.55 ± 0.04^b^	0.56	0.00 ± 0.00^b^	0.00	0.92 ± 0.79^b^	0.65
(*E*)‐2‐Hexenal	1224	99.43 ± 3.69^a^	59.79	7.57 ± 9.45^b^	7.77	35.47 ± 10.53^c^	26.76	23.38 ± 7.96^bc^	16.53
(*E*,* E*)‐2,4‐Hexadienal	–	3.63 ± 0.15^a^	2.18	0.00 ± 0.00^b^	0.00	1.68 ± 0.49^c^	1.26	1.41 ± 0.29^c^	1.00
Benzaldehyde	1536	1.77 ± 0.15^a^	1.06	3.13 ± 2.71^a^	3.21	3.47 ± 0.52^a^	2.62	1.90 ± 1.13^a^	1.35
*p*‐Tolualdehyde	1665	2.95 ± 0.60^a^	1.77	6.99 ± 0.41^b^	7.18	7.00 ± 1.15^b^	5.28	5.64 ± 1.05^b^	3.99
	Subtotal	**113.14**	**68.03**	**18.24**	**18.72**	**47.61**	**35.92**	**33.25**	**23.52**
Esters									
Methyl butanoate	–	9.56 ± 1.30^a^	5.75	0.00 ± 0.00^b^	0.00	0.00 ± 0.00^b^	0.00	0.00 ± 0.00^b^	0.00
Methyl hexanoate	–	7.81 ± 0.29^a^	4.70	0.00 ± 0.00^b^	0.00	2.58 ± 0.68^c^	1.95	2.35 ± 0.80^c^	1.66
Methyl 2‐hexenoate	–	0.48 ± 0.15^a^	0.29	0.22 ± 0.05^a^	0.23	0.27 ± 0.02^a^	0.20	0.28 ± 0.03^a^	0.20
Methyl heptanoate	–	0.21 ± 0.02^a^	0.13	0.00 ± 0.00^b^	0.00	0.00 ± 0.00^b^	0.00	0.00 ± 0.00^b^	0.00
Methyl benzoate	–	10.45 ± 0.59^a^	6.29	6.04 ± 1.29^b^	6.20	9.02 ± 1.68^ab^	6.80	9.10 ± 0.87^ab^	6.43
Methyl salicylate	–	0.41 ± 0.08^a^	0.25	0.29 ± 0.02^a^	0.30	0.39 ± 0.10^a^	0.29	0.37 ± 0.06^a^	0.26
Methyl N‐methyl anthranilate	–	1.15 ± 0.11^a^	0.69	0.69 ± 0.25^a^	0.71	0.00 ± 0.00^b^	0.00	0.70 ± 0.35^a^	0.50
Methyl anthranilate	–	0.38 ± 0.01^a^	0.23	0.34 ± 0.01^a^	0.35	0.35 ± 0.08^a^	0.27	0.27 ± 0.01^a^	0.19
Hexyl acetate	1268	0.38 ± 0.10^a^	0.23	0.00 ± 0.00^b^	0.00	0.00 ± 0.00^b^	0.00	0.00 ± 0.00^b^	0.00
2‐Hexenyl acetate	–	5.38 ± 0.43^a^	3.23	0.66 ± 0.05^b^	0.68	1.17 ± 0.64^b^	0.89	0.70 ± 0.24^b^	0.50
Ethyl butanoate	1037	1.30 ± 0.11^a^	0.78	0.00 ± 0.00^b^	0.00	0.58 ± 0.14^b^	0.44	0.69 ± 0.05^b^	0.49
Ethyl benzoate	–	0.70 ± 0.15^a^	0.42	0.43 ± 0.08^a^	0.44	0.53 ± 0.09^a^	0.40	0.61 ± 0.11^a^	0.43
	Subtotal	**38.22**	**22.99**	**8.67**	**8.91**	**14.89**	**11.24**	**15.09**	**10.66**
Ketones									
Diacetyl		0.00 ± 0.00^a^	0.00	26.22 ± 13.81^ab^	26.91	33.99 ± 6.66^b^	25.64	34.12 ± 11.40^b^	24.12
Acetoin	1298	0.00 ± 0.00^a^	0.00	7.02 ± 3.85^ab^	7.21	2.72 ± 0.93^ab^	2.05	13.84 ± 7.26^b^	9.78
6‐Methyl‐5‐hepten‐2‐one	1340	0.40 ± 0.07^a^	0.24	0.51 ± 0.07^a^	0.52	0.42 ± 0.11^a^	0.32	0.45 ± 0.03^a^	0.32
2‐Nonanone		3.26 ± 0.37^a^	1.96	6.04 ± 0.47^b^	6.20	3.59 ± 2.38^ab^	2.71	4.36 ± 0.36^ab^	3.08
	Subtotal	**3.65**	**2.20**	**39.79**	**40.84**	**40.72**	**30.72**	**52.77**	**37.30**
Terpenes									
Myrcene	1150	0.61 ± 0.20^a^	0.37	0.38 ± 0.06^a^	0.39	0.44 ± 0.06^a^	0.34	0.42 ± 0.04^a^	0.30
	Subtotal	**0.61**	**0.37**	**0.38**	**0.39**	**0.44**	**0.34**	**0.42**	**0.30**
	Total	**166.28**		**97.42**		**132.53**		**141.47**	

*Notes*

L10: *Lactobacillus helveticus* L10; L26: *Lactobacillus paracasei* L26; HN001: *Lactobacillus rhamnosus* HN001.

^a,b,c^Statistical analysis at 95% confidence level with same letters indicating no significant difference.

^d^Experimentally determined LRI on the DB‐FFAP column, relative to C8‐C40 hydrocarbons.

^&^RPA: relative peak area=100 x (peak area/total)

Malic acid was the most abundant organic acid in fresh star fruit juice (Table 1). It was significantly reduced from 3.5 g/L to around 1.9–2.0 g/L in all fermentations (Table 1). This could be largely attributed to malolactic reaction by decarboxylation of malic acid to lactate (Schümann et al., 2013). In fact, most lactobacilli could decarboxylate malic acid directly into lactic acid by a single malolactic enzyme (Hutkins, 2007).

Lactic acid was the major acid produced during fermentation (Table 1). *L. rhamnosus* produced significantly higher level of lactic acid (4.4 g/L) than that of *L. helveticus* (3.43 g/L) and *L. paracasei* (3.70 g/L) (Table 1) in correlation with the pattern of sugar consumption (Figure 3) and pH reduction (Figure 2b). As mentioned earlier, malic acid could be one of the major sources for the accumulation of lactic acid. However, the major pathway for lactic acid production in this study should be from the transformation of a hexose into two pyruvic acids through the Embden–Meyerhof pathway, followed by the reduction in pyruvic acid into lactic acid by NAD^+^ dependent dehydrogenases (Lengeler, Drews, & Schlegel, 2009), as all the lactobacilli used are homofermentative.

Similar but trace amounts of α‐ketoglutaric acid (0.06 g/L) were produced in all star fruit juices fermented by different probiotic strains. α‐Ketoglutaric acid could be formed from the catabolism of glutamic acid (Thage et al., 2004). Oxalic acid remained stable during fermentation (Table 1). This indicated that probiotics used in this study would not be able to degrade the oxalic acid in star fruit juice fermentation at 30°C. Oxalic acid is undesirable due to its ability to form salts of oxalic acid that may cause kidney stones.

### 3.5 Changes in volatile profiles

Volatiles in star fruit juice before and after fermentation including acids, alcohols, aldehydes, esters, ketones, and terpenes are summarized in Table 2. The different probiotic strains resulted in drastic variations of the volatiles in star fruit juice beverages (Table 2).

The most abundant volatile group in fresh star fruit juice was aldehydes, which constituted relative peak area (RPA) of 68.03% (Table 2). However, after fermentation, aldehydes (e. g. 1‐hexanal, (*E*)‐2‐hexenal, and (*E, E*)‐2,4‐hexadienal) were significantly degraded to low or trace levels (Table 2). *Lactobacillus helveticus* showed a higher ability in the conversion of aldehydes with (*E*)‐2‐hexenal and 1‐hexanal being decreased by 13.13‐fold and 9.76‐fold, respectively, while (*E, E*)‐2,4‐hexadienal was totally consumed after fermentation (Table 2). In comparison, *L. paracasei* and *L. rhamnosus* only resulted in 2.80‐ and 4.25‐fold reduction of (*E*)‐2‐hexanal and 2.16–2.57‐fold reduction of (*E, E*)‐2,4‐hexadienal (Table 2). The degradation of these odorous (green, grassy) aldehydes could be attributed to the redox balance to produce the corresponding alcohols (Blagden & Gilliland, 2005).

On the other hand, the aldehydes including benzaldehyde and tolualdehyde that were perceived as nutty and almond‐like aroma notes were increased after fermentation, with higher amounts produced by *L. helveticus* and *L. paracasei* (Table 2). These compounds may be derived from the aromatic amino acids such as phenylalanine via the aminotransferase reaction (van Kranenburg et al., 2002).

The second most abundant volatiles in fresh star fruit juice were esters (methyl and ethyl esters, acetate esters), contributing to 22.99% of total peak area (Table 2). All endogenous esters except for methyl benzoate were significantly degraded to trace or undetectable levels after fermentation (Table 2). *Lactobacillus helveticus* showed the highest ester degradation compared with the other two strains (Table 2). It is interesting to note that the short‐chain esters (methyl butanoate, ethyl butanoate, n‐hexyl acetate, and methyl heptanoate) were degraded more drastically compared to the long‐chain esters (e.g., methyl salicylate and methyl anthranilate) (Table 2). Our results agreed with the findings of Bintsis, Vafopoulou‐Mastrojiannaki, Litopoulou‐Tzanetaki, and Robinson (2003), in which most *Lactobacillus* strains, especially *L. acidophilus,* exhibited high esterase activities, which were involved in the breakdown of short‐chain fatty acid esters.

Ketones were the largest volatile group produced in all fermentations with the increase of RPA from 2.20% to 30.72%–40.84% (Table 2). Diacetyl and acetoin were the major ketones that were produced with the highest production in star fruit juice fermented with *L. rhamnosus* (Table 2). The production of diacetyl and acetoin by the probiotic lactobacilli was well documented (Benito de Cárdenas, Ledesma, Pesce de Ruiz Holgado, & Oliver, 1985; Liu, Holland, & Crow, 2003). These two buttery aroma compounds could be derived from the serine catabolism (Liu et al., 2003) or from citric acid (Hugenholtz, 1993). On the other hand, *L. helveticus* was found to be a good producer of 2‐nonanone (contributing fruity and musty odor) compared with the other two probiotic strains (Table 2). This was in line with the findings in probiotic fermented coconut water (Lee et al., 2013).

Alcohols were the second largest volatile group produced after probiotic fermentation (Table 2). *Lactobacillus paracasei* and *L. rhamnosus* produced higher levels of 1‐hexanol, (*E*)‐2‐hexen‐1‐ol, and linalool than those of *L. helveticus* (Table 2), and similar amounts of isoamyl alcohol, 1‐octen‐3‐ol, and 2‐ethylhexanol were produced in all fermentations (Table 2).

The increases in fresh, sweet green like C_6_ alcohols such as 1‐hexanol and (*E*)‐2‐hexen‐1‐ol may be due to the reduction in corresponding C_6_ aldehydes, as a reflection of the *Lactobacillus* in maintaining the redox balance (Budinich et al., 2011). In addition, these C_6_ alcohols could also be produced by hydrolyzing the hexenyl and hexanyl esters during fermentation as discussed above.

Isoamyl alcohol could be derived from leucine via amino acid metabolism and is commonly found in foods fermented by *Lactobacillus* (Thage et al., 2004). Linalool, which gives rise to the citrus and floral aroma in star fruit, was increased in fermented juice by *L. paracasei* and *L. rhamnosus* but not *L. helveticus* (Table 2). This observation was in agreement with the fermentation of probiotic coconut water, in which *L. helveticus* did not produce linalool after fermentation (Lee et al., 2013). On the other hand, the production of 1‐octen‐3‐ol and 2‐ethylhexanol could be derived from the oxidation of linoleic and linolenic acids (Broadbent et al., 2004). These two compounds could contribute to the mushroom‐like and sweet fruity‐like aroma notes to the star fruit juice beverages, respectively.

Volatile fatty acids (VFAs) were another important volatile group produced after star fruit juice fermentation (Table 2). These acids were mostly derived from the hydrolysis of esters or from sugars, organic acids, and amino acids. The increase in hexanoic acid and (*E*)‐2‐hexenoic acid corresponded to the decrease in 1‐hexanal and (*E*)‐2‐hexanal (Table 2), indicating the 6‐carbon aldehydes could be oxidized into their corresponding volatile acids by the *Lactobacillus*. The higher production of acetic, hexanoic, and (*E*)‐2‐hexenoic acids in star fruit juice fermented with *L. helveticus* could be explained by its higher hydrolytic activity of the corresponding esters.

### 3.6 Principal component analysis of star fruit juice beverages

**Figure 4:**
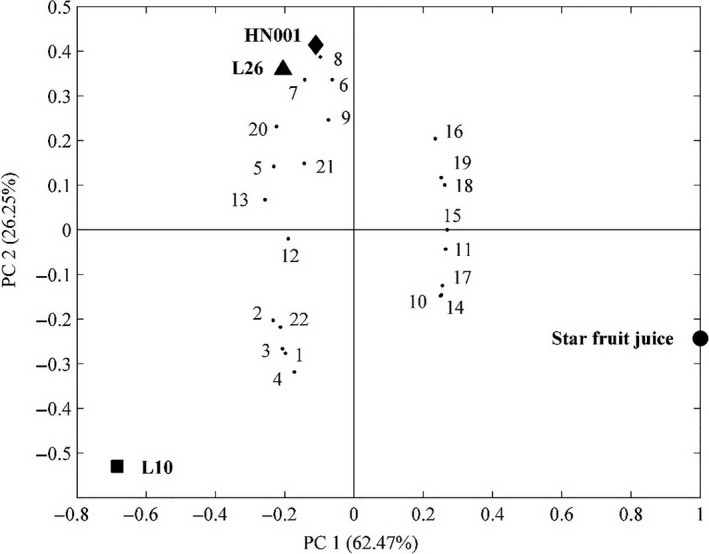
Biplot of principal component analysis of selected volatile compounds in star fruit juice and star fruit juice beverages. Star fruit juice (●); L10: *Lactobacillus helveticus* L10 (■); L26: *Lactobacillus paracasei* L26 (▲); HN001: *Lactobacillus rhamnosus* HN001 (♦). (1) Acetic acid, (2) hexanoic acid, (3) (*E*)‐2‐hexenoic acid, (4) decanoic acid, (5) isoamyl alcohol, (6) 1‐hexanol, (7) (*E*)‐2‐hexen‐1‐ol, (8) linalool, (9) 1‐nonanol, (10) 1‐hexanal, (11) (*E*)‐2‐hexenal, (12) benzaldehyde, (13) *p*‐tolualdehyde, (14) methyl butanoate, (15) methyl hexanoate, (16) methyl benzoate, (17) 2‐hexenyl acetate, (18) ethyl butanoate, (19) ethyl benzoate, (20) diacetyl, (21) acetoin, (22) 2‐nonanone

The selected 22 volatile compounds were subjected to principal component analysis (PCA) to discriminate the common characteristics and illustrate the variety of the volatiles among different fermentations (Figure 4). The first principal component (PC1) and the second principal component (PC2) accounted for 62.47% and 26.25% of the total variance, respectively. The star fruit juice in the positive part of PC1 was segregated due to the high contents of some aldehydes (e.g., 1‐hexanal and (*E*)‐2‐hexenal), methyl esters (methyl esters of butanoate, hexanoate and benzoate), ethyl esters (ethyl esters of butanoate and benzoate), and 2‐hexenyl acetate (Figure 4). The star fruit beverage fermented with *L. paracasei* and *L. rhamnosus* in the second quadrant was separated due to their high contents of alcohols (isoamyl alcohol, 1‐hexanol, (*E*)‐2‐hexen‐a‐ol, linalool and 1‐nonanol), ketones (diacetyl and acetoin), and *p*‐tolualdehyde (Figure 4), while the star fruit beverage fermented with *L. helveticus* was distinguished from the other two probiotic strains and star fruit juice by its high contents of fatty acids (acetic, hexanoic, (*E*)‐2‐hexenoic, and decanoic acid), benzaldehyde, and 2‐nonanone.

## 4 CONCLUSIONS

The potential of three different probiotic lactobacilli to ferment star fruit juice was evaluated, and the results showed that all three lactobacilli were able to grow well with final cell counts of around 10^8^ CFU/ml. The highest level of lactic acid was produced by *L. rhamnosus*, resulting in the significantly lower pH of star fruit juice beverage than the juices fermented with *L. helveticus* and *L. paracasei*. Endogenous volatile compounds in star fruit juice were degraded to low or undetectable levels, while new volatile compounds including ketones, alcohols, and fatty acids were produced by different probiotic strains at varying levels, contributing flavor complexity to the beverage. Therefore, the findings suggest that probiotic strains can be used to develop novel nondairy functional star fruit juice beverage with different flavor notes.

## CONFLICTS OF INTERESTS

All authors declare that they do not have conflicts of interests.
